# Test-enhanced learning: analysis of an experience with undergraduate nursing students

**DOI:** 10.1186/s12909-015-0464-5

**Published:** 2015-10-24

**Authors:** Linda Messineo, Manuel Gentile, Mario Allegra

**Affiliations:** Institute for Educational Technologies, National Research Council, CNR-ITD, Via Ugo La Malfa 153, Palermo, 90146 Italy

**Keywords:** Test-Enhanced Learning, Undergraduate nursing students, Psychology education, Test anxiety

## Abstract

**Background:**

This study is based on the evidence that tests can be used as an educational tool to enhance learning, not just as an evaluation tool. There is a growing body of research that shows that participating in repeated testing improves learning, a phenomenon defined as Test-Enhanced Learning. The aim of the present study was to analyse the effect of the use of a test enhanced learning program integrated into a general psychology course for undergraduate nursing students and its interaction with the students’ test anxiety.

**Methods:**

161 undergraduate nursing students attending a General Psychology course followed an educational program based on Test-Enhanced Learning methodology. Students were divided into two groups, an experimental group (TEL group) and a control group (Re-study group). TEL students took a multiple-choice test on the lecture topics. The Re-study group just read study material. Testing and re-study occurred at intervals of about a week after each lesson. TEL students received feedback immediately after each test. About two weeks after the end of the lessons, all the students took a final cumulative test on all the topics. Statistical analysis was used to analyse students’ performances. After the administration of the cumulative unit test, all the students took a graded examination.

**Results:**

Students in the TEL group performed better than the controls, both in the final cumulative test and in a graded examination. TEL participants experienced better final cumulative test results than students not tested (*M*_*TEL*_ = 23.11, *M*_*Re-study*_ = 20.47, *t*(109.86) = −2.57, *p <* 0.05*, r* = 0.24). Test-Enhanced Learning program participation has a positive impact on exam performance (β_G_Step1_ = 0.46, *p* < 0.001). Finally, the analysis performed shows a slight moderating effect of test anxiety on Test-Enhanced Learning (β_GxTA_Step3_ = 0.15, *p* < 0.05).

**Discussion and Conclusions:**

Test-Enhanced Learning can be an effective tool for promoting and enhancing learning. In fact, taking tests after studying produced better long-term retention and then better final test performance than re-reading without testing.

Both students in the TEL group and the Re-study group with a high test anxiety level perform less well than colleagues with lower test anxiety. Nevertheless, students with higher test anxiety may obtain more benefits from participating in a Test-Enhanced Learning process than people with lower test anxiety.

Further studies on larger and more representative samples are necessary in order to investigate the effect of test anxiety on Test-Enhanced Learning.

## Background

In educational settings, tests are commonly used to assess students’ learning. Several studies in cognitive psychology, conducted in laboratories over the past decades, have consistently shown that repeated testing on topics studied produces considerable improvement in later tests compared to re-studying the same contents [1] (for reviews, see [2, 3]). Tests can therefore represent not only an assessment tool of acquired knowledge, but can also change the process of learning and significantly improve long-term retention of contents through the promotion of the process of retrieval and retention of information. These factors facilitate learning and memorizing of the study material tested. This phenomenon is known as Test-Enhanced Learning (TEL) or testing effect. The benefits of taking a test to enhance performance in a later test, similar to the initial test, were studied under a wide range of different conditions (such as free or cued recall), using different materials (such as paired word or picture associated), different learners (such as children, young and adults), and different types of test (such as open or multiple choice tests) [4–11].

Research has also shown that, after taking a test, learners improve their ability to encode information, to reflect on and self-assess their acquired knowledge related to material studied and show an improvement in long-term retention.

Despite the large amount of research carried out in psychology laboratories, the study of the testing effect in educational settings, or with material similar to that used in educational settings, has taken place more recently.

In the last few years, studies of this procedure in real or simulated educational settings, especially in schools, have been conducted by [12–18]. Nevertheless, a better understanding of how certain psychological student characteristics (e.g. test anxiety) may affect the implementation of Test-Enhanced Learning in the classroom is needed. For example, in a laboratory setting, it was observed that the benefit of the repeated test in undergraduate students with low working memory capacity and with high test anxiety was very small [19]. Several research studies show that high levels of test anxiety generally produce a decrement in students’ learning and performance [20]. Students with high test anxiety feel tense in evaluative situations [21, 22] and commonly obtain poorer test scores and grades [23–25].

In order to improve and enhance the learning process through the use of repeating testing, it is important to better understand how tests can be integrated into real educational contexts and to clarify whether test anxiety may affect retrieval practice benefits.

### Research hypotheses

The aim of the current study was to examine the effect of a TEL program on long-term retention and its interaction with students’ test anxiety in a General Psychology course for undergraduate nursing students.

The main research hypothesis was that an educational program based on the principles of TEL enhances later retention of topics studied during traditional lectures. Participation in TEL activities should lead to an improvement in learning performance both in a final cumulative test (H1) and in the graded performance in a final examination (H2).

The third hypothesis was that test anxiety has an effect on performance in the graded examination (H3).

Finally, the fourth hypothesis was that the TEL effect on the examination results is moderated by anxiety; students with high test anxiety should benefit more than students with lower test anxiety (H4).

## Methods

### Participants

According to the local ethical policy, no formal approval by the Ethics Committee was necessary. We clearly formulated an experimental design which we communicated to the Faculty board of the University of Palermo assuring that ethical standards were met, in compliance with the Declaration of Helsinki, and in accordance with the Ethical Code of the University of Palermo (Rector's Decree no. 400/2012), the Psychology Ethical Code and the Italian Personal Data Protection Code (Leg. Decree no. 196/2003). In particular, the study respected the following aspects: students were asked to provide voluntary written informed consent before participation; students could decide to interrupt their participation at any moment; personal data protection was ensured; there was no conflict of interest.

Subjects were undergraduate nursing students attending a course of General Psychology at the University of Palermo.

The original sample consisted of 201 students. Students were randomly assigned to perform different activities. One hundred and four students were assigned to the experimental group (TEL group), while the control group (Re-study group) was composed of ninety seven students. At the beginning of the TEL program, a number of students in the Re-study group (40 participants) were excluded from the sample because some data was not recorded properly due to a technical problem with the web system implemented for the research study.

The sample of students effectively involved in the study consisted of 161 students (102 females and 59 males, aged from 18 to 31 years). All the statistical analysis was carried out taking into account the unequal sample size of the groups. The following table (Table 1) reports the gender and the age distribution of the two groups.

**Table 1:** Demographic characteristics of nursing students by group

	Females (*n*)	Males (*n*)	Age
Re-study group *n* = 57	34	23	*M* = 20.65, *SD* = 2.28
TEL group *n* = 104	68	36	*M* = 20.72, *SD* = 2.82
Total participants *N* = 161	102	59	*M =* 20.70, *SD* = 2.63

### Materials and design

The study involved four phases, in each of which different data were collected. The study lasted two months.

A web-platform was implemented for the experimentation. We activated different user profiles for students in the experimental group and students in the control group.

The first measurement (session 0) took place before starting the TEL program and all students were asked to complete the same questionnaire. Specifically, we measured some demographic aspects as well as test anxiety using the Test Anxiety subscale of the Motivated Strategies for Learning Questionnaire. In the second phase (sessions 1–8), TEL students participated in the TEL program taking tests, and the Re-study group read re-study material. Testing and re-study occurred at intervals of about one week after traditional teaching of a specific topic. The TEL component of the course consisted in a series of multiple-choice tests, which students in the experimental group had to take after their weekly lessons in General Psychology. The activities for the experimental group were not mandatory.

In the third phase (session 9), two weeks after the end of the lessons, all the students took a final cumulative test on the content of each lecture. In the final phase (session 10), the grades of all the students in the final examination were measured.

Finally, in order to compensate for the expected TEL benefit, appropriate procedures of compensation were designed for the students in the Re-study group (e.g. the integration of curricular activities at the end of the TEL program).

The research phases listed above are described in the following paragraphs. Table 2 shows the scheduling of the experimental design with an indication of the different conditions used in the experiment in the different sessions.

**Table 2:** Experimental sessions

Group	Session 0	Session 1-8	Session 9	Session 10
Re-study	Pre-test	Re-study	Final cumulative test	Final exam
TEL	Pre-test	TEL	Final cumulative test	Final exam

The experiment spanned all the weeks of the course and continued for a further two weeks after the course for the final cumulative test.

### Phase one: Demographic aspects and test anxiety

In the first phase, all students accessing the platform for the first time completed a personal data questionnaire as well as the Motivated Strategies for Learning Questionnaire to measure test anxiety. The personal questions were concerned with basic demographic information such as gender and age. Students were also asked if they had followed other university courses and if they had already attended a general psychology course. Students who had already studied psychology were excluded from the sample. Test anxiety was measured according to the following definition: “the set of phenomenological, physiological and behavioural responses that accompany concern about possible negative consequences or failure on an exam or similar evaluative situation” [26].

To measure students’ test anxiety, we used the Italian version of the Motivated Strategies for Learning Questionnaire (MSLQ) [27, 28]. MSLQ is a self-report questionnaire developed to evaluate college student motivation and learning strategies. It consists of 81 items on a 7-point Likert-scale, and it is divided into two components. The first section, consisting of 31 items, assesses the motivational orientation of students, while the second section evaluates the students’ learning strategies. Each of the sections is composed of different scales, which can be used separately or together according to the aim of the research. One subscale of the motivational scale is the test anxiety scale and this was administered to all students, in both the experimental and control groups, during the initial phase of the experimentation. The Test Anxiety subscale rates students on two subcomponents: a cognitive component, related to negative thoughts that disturb their performance, and an emotional component that activates affective and psychological aspects. These components are the best predictors of a decrease in performance in an exam [27, 28].

### Second phase: test about the lecture

The General Psychology course in the nursing degree lasted two months and consisted of weekly traditional type lectures. The course in General Psychology aims to introduce students to the scientific study of mental and behavioural human processes. All the students participated in the psychology course covering the basic subjects in general psychology. For the experimentation, only the following eight topics were selected: attention, sensation, perception, motivation, emotions, learning, memory, cognition and intelligence. For each of these eight psychology topics, two groups of 10 multiple choice questions (with 4 possible answers) were defined. One set of questions was used for the definition of the test used during the TEL program (experimental group) and another set of questions was used for the final cumulative test (experimental and control group). After each weekly lesson, dealing with a single issue, an email was sent to students with instructions on how to use the TEL system. Emails were different for the experimental group and the control group. The experimental group took a weekly session test on topics studied during the weekly lecture, while the control group re-read the content of the lecture. Test was active for a limited time of three days from activation, and students were required to take the test outside lesson times. Students could take a test only once, and there was a time limit for its completion. Immediately after the test, the system provided feedback on the correctness or incorrectness of all the answers. Feedback included all items with all answer options, and the answers given by the students were highlighted. If the answer was correct, the question-answer pair was highlighted in green, and a message “correct answer” appeared. If the answer was incorrect, the question-answer was highlighted in red, and the correct answer in green and the message “incorrect answer” appeared. Feedback is a crucial element in increasing the power of testing in terms of retention boosting [29, 30] and, moreover, research has shown that providing delayed feedback for all the questions at the end of the whole test, is better than giving immediate feedback after each individual answer [31–33].

The control group accessed a section in which they read question-answer pairs. Every time a re-study student accessed material, after reading the entire set of question-answer pairs, he had to click on “I have read the topic” and then concluded the study session. The learning platform was designed to allow tracking of all the students’ actions.

### Third phase: final cumulative test

Two weeks after the end of the course (session 9), all students in both the experimental group and the control group completed a final cumulative test of eighty questions, on the eight psychology topics, similar to items used during the TEL program.

### Fourth phase: grade in the final examination

Eight weeks after the experimentation (session 10), following the administration of the cumulative unit test, all the students took the final exam and their grades were analysed. The final exam was an oral exam, covering all the content already studied during the course.

## Results

A Welch’s *t*-test was performed to confirm Hypothesis 1. According to Welch [34], this test is more reliable than a Student’s *t*-test when the two samples have unequal variances and unequal sample sizes. Moreover, the effect size of the TEL was calculated.

Following Cohen & Cohen’s approach [35], moderated regression analysis was used for testing interaction effects. Hierarchical multiple regression analyses were performed to detect the main effects of testing effect and interaction effects with test anxiety as the moderator variable.

In order to test interaction effects, a multiplicative term was created for the standardised independent variables [36, 37].

The standardised independent variables were introduced into the equation in three successive steps [38].

In the first step, the testing condition was introduced to check the influence of TEL participation on the final cumulative test score as well as exam performance. Next (step 2), the moderator variable (test anxiety), and finally (step 3) the two-way interaction (testing condition x moderator) were added. The significant interaction effects would support Hypothesis 4.

However, we also took into account the main effects, given that, as Jaccard et al. [38] point out, the main effects of the independent variables generally constitute significant information. The significant effect of the moderator variable supports Hypothesis 3. The changes in *R*^*2*^ in the moderator model would support Hypothesis 4, suggesting that test anxiety moderates the link between testing conditions and the final cumulative test.

In order to interpret the standardised variables a priori, unstandardized regression coefficients (B) [37] are presented in Tables 3.

**Table 3:** Results of the moderated regression analysis

		*R*	*R* ^*2*^	ΔR^2^	B	B SE	β
Step1		0.21***	0.21				
	Intercept				12.68***	0.69	
	Group				5.37***	0.85	0.46
Step2		0.28***	0.27	0.07***			
	Intercept				12.57***	0.66	
	Group				5.55***	0.82	0.48
	Test anxiety				−1.44***	0.39	−0.26
Step3		0.30***	0.28	0.02*			
	Intercept				12.47***	0.65	
	Group				5.62***	0.81	0.48
	Test anxiety				−2.63***	0.71	−0.47
	Group × Test anxiety				1.71*	0.85	0.15

*Note.* *p < 0.05, **p < 0.01, ***p < 0.001

The final test consisted of 10 multiple-choice questions on each of the 8 topics tested and re-studied during the TEL activity. The questions in the final test and the exam were not the same as those used in testing during the TEL program. On average, TEL participants experienced better (*M*=23.11, *SE*=0.59) final cumulative test scores than non-tested students (*M*=20.47, *SE* = 0.84). This difference is significant, *t*(109.86) *= −*2.57*, p* < 0.05; it represents a medium sized-effect, *r* =0.24 (H1).

Twelve students that completed the study did not take the examination; they were excluded from the sample in order to verify the H2, H3 and H4 hypothesis. Therefore, data from 149 students were used.

The MSLQ Test Anxiety subscale consisted of 5 items (α = 0.76). The results of the regression analysis are reported in Table 3. The aim of the first model was to verify the TEL effect on the examination results.

Group (β_G_Step1_ = 0.46, *p* < 0.001) was a significant predictor of exam performance. The overall model fit was *R*^*2*^ = 0.21 (H2).

In the second step of Cohen & Cohen’s approach, Group (β_G_Step2_ = 0.48, *p* < 0.001) and Test Anxiety (β_TA_Step2_ = -0.26, *p* < 0.001) were significant predictors of exam performance. The overall model fit was *R*^2^ = 0.27 and also ΔR^2^ = 0.07, *p* < 0.001 highlighting a better fit of the second model (H3).

Finally, the overall model fit of the third model was *R*^*2*^ = 0.28; ΔR^2^ = 0.02, *p* <0.05 which highlights a slight improvement. Group (β_G_Step3_ = 0.48, *p* < 0.001) and Test Anxiety (β_TA_Step3_ = -0.47, *p* < 0.001) and the interaction factor Group × Test Anxiety (β_GxTA_Step3_ = 0.15, *p* < 0.05) were all significant predictors of exam performance (H4).

**Fig. 1:**
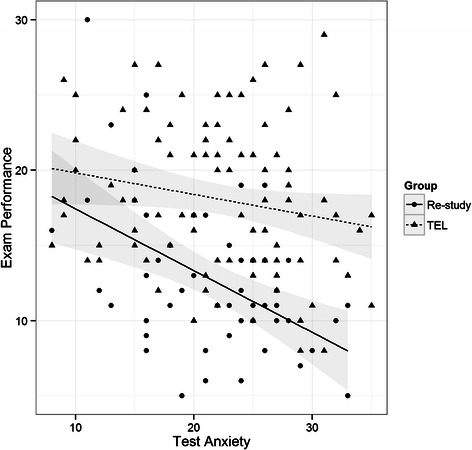
Relationship between the exam performance variable and the test anxiety

In Fig. 1, the relationship between the exam performance variable and the test anxiety variable is shown. The dots represent the position of observations from the Re-study group, while the triangles are the observations for the TEL group. For each group a regression line and its CI is reported. Both lines show a negative trend in exam performance for increasing test anxiety. Nevertheless, the slope for the TEL group relation is less steep than the slope for the Re-study group in absolute terms, suggesting a minor effect of test anxiety on TEL students.

**Fig. 2:**
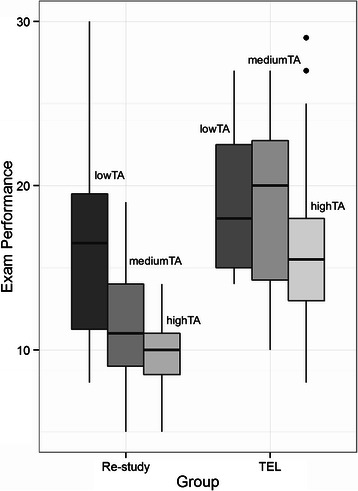
Testing Effect at relatively low, medium and high levels of the test anxiety

Table 4 shows the effect of the TEL program on the students at three different levels of test anxiety (TA). Low TA group includes students with values < (mean - *SD*), medium TA group includes students in the interval (mean ± *SD*), and high TA group includes students with values > (mean + *SD*). In the Table 4, the number of students in each condition (*n*) is also indicated. Figure 2 shows the boxplots for each group.

**Table 4:** Statistics about distribution of students (Re-study and TEL) at relatively low, medium and high levels of the test anxiety

	Exam performance		
	Low TA	Medium TA	High TA
Re-study group	*n* = 14	*n* = 29	*n* = 10
	*M* = 16.43, *SD* = 6.49	*M* = 11.86, *SD* = 3.74	*M* = 9.8, *SD* = 2.57
TEL group	*n* = 20	*n* = 46	*n* = 30
	*M* = 19.05, *SD* = 4.29	*M* = 18.80, *SD* = 4.94	*M* = 16.23, *SD* = 5.16

## Discussion and Conclusions

The results of this study demonstrate that using tests enhances learning and has a significant effect on improving long-term retention of the different topics studied.

These results are consistent with other studies carried out in this area, which have shown that testing has considerable benefits for memory retention. In cognitive psychology this effect is referred to as the “testing effect”. The majority of initial studies of this effect were carried out in psychology laboratories with the use of non-significant material of different kinds (e.g. verbal and visual material). Despite the large number of studies in this area, it is only recently that studies have been made into the possibility of using this procedure in education. These studies have shown that it is possible to use this methodology to improve memory in real educational settings. The testing effect is a robust effect and even with fairly small samples, as in the case of our study, it is possible to observe the benefits of using tests.

Therefore, TEL methodologies could certainly be an effective tool for promoting and enhancing learning. In fact, taking tests after studying produced better long-term retention followed by better final test performance than re-reading without testing.

This study shows that both students in the TEL group as well as those in the Re-study group with a high test anxiety level perform less well than colleagues with lower test anxiety. Nevertheless, students with higher test anxiety may obtain more benefits from participating in a TEL learning process. A slight moderating effect of test anxiety on TEL was observed. However, further investigation is necessary in order to improve the understanding of test anxiety - TEL program interaction.

One limitation of the current study is that a large number of students in the Re-study group were excluded from the sample (40 participants) because some data was not recorded properly due to a technical hitch with the web system implemented for the research study.

The lack of measurement of test anxiety close to the final cumulative tests and the final examination represents another limitation of this study, but at the same time a way to further investigate the relationship between TEL and test anxiety.

In conclusion, new studies for better understanding how a TEL program can be integrated into real contexts are needed. In an interesting recent study, Hinze and Rapp investigated the effects of performance pressure on retrieval practice [39]. They found that only low-stake tests led to performance benefits over a re-study control group. A possible next step of particular relevance in this research area could be to replicate our study in low-stake and high-stake situations, in which, for example, performance pressure during test is induced, and investigate how test anxiety may affect retrieval practice benefits in the two different situations.
